# Applying user-centered design to develop a culturally sensitive, low-calorie meal plan for enhancing dietary behavioral control in MASLD

**DOI:** 10.1186/s40795-026-01347-8

**Published:** 2026-05-06

**Authors:** Maya Balakrishnan, Paola Martinez, Brett Deng, Ivonne Arguelles, Crystal Arguelles, Terri L. Fletcher, Natalia I Heredia, Myriam Ibarra, Anna Christine Rome

**Affiliations:** 1 Center for Innovations in Quality, Effectiveness and Safety, Michael E. DeBakey Veterans Affairs Medical Center, Houston, TX USA; 2 Department of Internal Medicine, Section of Gastroenterology and Hepatology, Baylor College of Medicine, One Baylor Plaza, Houston, TX 77030 USA; 3 Menninger Department of Psychiatry and Behavioral Sciences, Baylor College of Medicine, Houston, TX USA; 4 VA South Central Mental Illness Research, Education and Clinical Center, Houston, TX USA; 5 UTHealth Houston School of Public Health, Houston, TX USA; 6 Outpatient Clinical Nutrition Services, Harris Health System, Houston, TX USA

**Keywords:** Fatty liver, Health status disparities; diet therapy, Dietary intervention, Cultural adaptation, Ecological momentary assessment

## Abstract

**Background:**

Dietary changes are essential for managing metabolic dysfunction-associated steatotic liver disease (MASLD), yet patients often face barriers related to knowledge, skills, cost, time, and cultural fit. Aims: The purpose of this paper is to report a process for culturally tailoring a dietary intervention and its application to create a calorie-restricted meal plan for Mexican and Central American patients with MASLD. Guided by the Theory of Planned Behavior and user-centered design, we produced a culturally tailored seven-day structured meal plan aligned with dietary guidelines for MASLD and weight loss.

**Methods:**

We conducted a three-phase, mixed-methods process among Mexican/Central American patients with MASLD from a safety-net healthcare system in Houston, Texas (*n* = 25). Phase 1 characterized meal patterns and preferences through semi-structured interviews. Phase 2 integrated findings with clinical nutrition guidelines to develop structured meal plan prototypes at 1200-, 1500-, and 1800-calorie levels. Phase 3 tested usability through ecological momentary assessments and daily interviews.

**Results:**

Participants typically consumed home-cooked meals centered on animal protein, legumes and simple grains, with lunch and dinner preparation being most challenging. Recipe modifications focused on increasing fiber and reducing fat and refined carbohydrates. Usability testing showed that participants found the plans culturally aligned and practical, improving portion awareness and dietary self-efficacy.

**Conclusions:**

This study offers a patient-centered process for culturally tailoring dietary interventions and its output, a calorie- restricted meal plan that shows preliminary feasibility and acceptability on user testing. Next steps (underway) are to evaluate the meal plan’s larger-scale implementation and impact on dietary change and weight loss.

**Supplementary Information:**

The online version contains supplementary material available at 10.1186/s40795-026-01347-8.

## Introduction

Metabolic-dysfunction associated steatotic liver disease (MASLD) is the most common chronic liver disease, affecting over 30% of adults in the United States [1, 2]. Caused by obesity and insulin resistance, it is closely related to metabolic syndrome and associated with cardiovascular complications and progressive liver injury that can culminate in cirrhosis and liver failure [3].

Dietary change is central to MASLD treatment. Sustained weight loss of 7–10% is associated with improvements in liver disease and cardiovascular outcomes [4]. To promote weight loss, clinical guidelines emphasize reducing total caloric intake. Guidelines also promote holistic dietary patterns, aligned with Mediterranean-patterned diets, given their independent associations with improved hepatic steatosis and metabolic markers [5]. Lastly, guidelines recommend reducing sugar-sweetened beverages, saturated fat, red and processed meats, and ultra-processed foods, given their associations with increased MASLD risk [6–9].

However, dietary change is difficult. It requires planning meals, modifying cooking practices, and consistently eating appropriate portion sizes. This requires a high degree of perceived behavioral control, which refers to how capable a person feels of achieving healthy eating behaviors and is a concept similar to self-efficacy. According to the Theory of Planned Behaviors, behavioral control is a key determinant of a motivation and adherence to dietary recommendations [10].

Patients with MASLD and related metabolic conditions frequently report barriers to behavioral control, including inadequate knowledge and skills for healthy eating, as well as challenges related to food cost, accessibility, and cultural or family eating practices [11–17]. Interventions have attempted to address these barriers using different strategies. Providing pre-prepared, portion-controlled meals [18, 19] or liquid meal replacements [20] reduces the need for individuals to apply dietary knowledge or skills in daily food choices, and improves weight loss rates in clincial trials. Alternatively, cooking classes and culinary medicine based interventions aim to build dietary knowledge, self-efficacy and practical skills [21]. These strategies, however, are resource-intensive and difficult to sustain long-term.

A potentially sustainable approach is the use of structured meal plans—dietary tools that specify what and how much to eat daily. These help translate complex nutritional recommendations into practical daily eating behaviors. Compared to standard dietary counseling alone, adding a structured five-day meal plan increased weight loss by 50% at 6 months and 100% at 1 year among adults in a clinical trial [22], and improved weight loss among adolescents with obesity [23]. Additional benefits include improved dietary knowledge, more accurate portion control, meal regularity, and reduced snacking.

To enhance behavioral control among Mexican and Central American (M/CA) patients with MASLD, we sought to develop a culturally tailored, seven-day structured meal plan. We prioritized this population due to its high representation in our Houston, Texas clinical setting and its disproportionate disease burden, with Hispanic adults having approximately 50% higher risk of MASLD and 42% higher risk of steatohepatitis compared with non-Hispanic adults [24]. The meal plan was conceptualized as a behavioral tool to model implementation of nutrition recommendations using familiar, culturally relevant foods [25].

In routine clinical care, structured meal planning is typically delivered through individualized counseling with a registered dietitian. However, this approach is difficult to scale given workforce limitations, time constraints, and cost barriers. As a result, a key translational gap persists: how to operationalize principles of clinical dietetics into practical, scalable approaches that clinicians, including those without formal dietetics training, can use to develop culturally appropriate dietary interventions for groups of patients?

To address this gap and clinical need, we developed a theory-informed, user-centered design approach – guided by nutrition science and the food literacy framework by Vidgen and Gellegos [26]. Our objective was translational: to operationalize principles of individualized dietetic counseling into a process that non-dietitian clinicians can use to develop culturally-tailored, group -based meal plans, reserving dietitians for components requiring specialized nutritional expertise. This paper describes the process and framework. We hypothesized that the process would yield a feasible, acceptable, culturally congruent meal plan that enhances behavioral control and healthy dietary habits.

## Methods

**Fig. 1:**
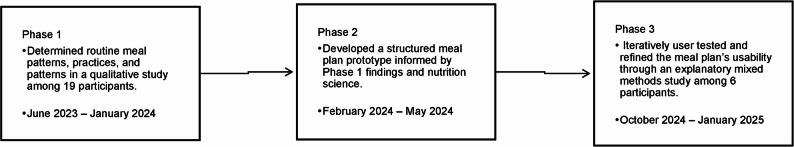
Three-phased user-center design process

To create the meal plan, we developed a three-phase, patient-centered design process (Fig. 1), adapted from user-centered design, which emphasizes iterative engagement with users to optimize product usability [27]. Our intended users were M/CA patients needing weight loss to manage MASLD, type 2 diabetes or metabolic syndrome. The study was IRB-approved by the relevant university and hospital systems and followed Helsinki Principles.

### Phase 1

Phase 1 was a qualitative study that used one-time semi-structured interviews to determine key features of across individual participants’ meal patterns, preferences, and practices that would influence meal plan construction for the broader group.

#### Study sample

We purposively sampled adults (18–70 years) with M/CA heritage, BMI ≥ 25 kg/m², and a diagnosis of MASLD, type 2 diabetes, and/or metabolic syndrome. Exclusions were advanced cirrhosis, hemoglobin A1C ≥ 9%, or serious competing comorbidities/contraindications to calorie reduction (ex advanced cancer, malabsorption disorders, pregnancy/breastfeeding, advanced renal disease). Recruitment occurred through diabetes and hepatology clinics and from among patients referred to dietitian led clinics for weight loss counseling within a safety-net health system in Houston, Texas. Clinicians introduced the study, and coordinators followed up with those interested. We obtained verbal consent and offered $20 for interviews. We anticipated needing 15–20 participants to reach data saturation, the point at which data obtained begins to be repetitive, given our study’s focused objectives, standardized interview questions and homogenous study population [28, 29].

#### Data collection

We developed a semistructured interview guide with input from dietitians experienced in counseling the priority population. It included questions, probes and prompts about cooking practices, meal composition, eating patterns and food preferences, as well as detailed accounts of meals consumed over four days (two weekdays, two weekend days), home-cooked recipes, and common household staples (Appendix A). The guide was pilot-tested and refined with feedback from internal staff and two volunteer prestudy participants.

Two bilingual female research assistants (CA, an undergraduate social science student, and MI, a Master’s-trained public health professional) conducted phone interviews in participants’ preferred language. Both were familiar with M/CA cuisines, qualitative interviewing, and had no prior relationship with participants.

Demographic and clinical data were collected via preinterview survey and electronic medical record review. MASLD, type 2 diabetes, hypertension and dyslipidemia were classified by documented diagnosis or treatment. Acculturation was measured using the Brief Acculturation Scale for Hispanics (score > 3 signifying a higher level of acculturation) [30] and food insecurity via the two-item Hunger Vital Sign (affirmative response to either indicating insecurity) [31].

#### Data analysis

We conducted thematic analysis to determine key features of participants’ meal preparation, eating practices and preferences that would influence meal plan construction [32]. Interviews were recorded, transcribed and translated from English to Spanish as needed and imported into Atlas.ti 23 (Atlas ti Scientific Software Development GmbH). The research team (MI, CA, BD, MB) reviewed the initial five transcripts to apply deductive codes based on interview questions and develop inductive codes from emerging content [33]. As no new codes emerged in subsequent interviews, we finalized the codebook and applied it to all transcripts. During weekly meetings, the team discussed transcripts and compared and resolved differences in coding. MB initially categorized and organized final codes in a thematic map, which the team iteratively refined. Final themes were structured around key domains important for creating a feasible meal plan. Rigor was maintained through review and discussion of transcripts, codes and themes.

### Phase 2

Phase 2’s objective was to create a prototype of the seven-day meal plan, guided by three main principles. The first was to ground the plan in patterns, practices and preferences emerging through phase 1 interviews. To achieve this, we structured the plan based on meal patterns and recipes frequently reported, and modified them using foods, ingredients and cooking equipment participants commonly had. Secondly, we constructed calorie-restricted meal plans to support average weight loss of one pound weekly. To this end, we planned one prototype rationed at three standard calorie levels: 1200, 1500, and 1800 calories per day for people with a baseline weight of 54 to 77, 78 to 99, and > 100 kg, respectively. These calorie thresholds have been used across several lifestyle weight-loss interventions, including the Diabetes Prevention and Look AHEAD Lifestyle Programs [34–36]. The third guiding principle was to align meals’ macronutrient distribution and composition with U.S. Department of Agriculture and World Health Organization dietary guidelines [37, 38], relevant for all metabolic syndrome conditions. To this end, we aimed for macronutrient distribution of 50% carbohydrates, 20% protein and 30% fat, with at least 25 g of dietary fiber per day. In addition, recipe modifications focused on incorporating complex carbohydrates (e.g., whole grains, lentils and legumes), lean protein sources (e.g., fish, chicken, beef), fresh vegetables and fruits, and plant-based oils. ACR, a licensed dietitian, modified recipes using *Nutritionist Pro™ Diet Analysis* software (Axxya Systems LLC, Redmond, WA).

### Phase 3

In phase 3, we assessed the meal plan’s usability among participants with surveys and interviews in a mixed-methods explanatory study design. The primary objective was to identify and fix major usability problems.

#### Study sample

We purposively recruited M/CA patients with BMI≥25 kg/m2 and MASLD who were not in Phase 1 and were willing to use the seven-day meal plan, applying Phase 1’s exclusion criteria and recruitment approach. The objective was iterative user testing, refining the prototype after each participant and concluding data collection when two consecutive participants reported no major usability problems. Based on published user testing guidance—indicating five users can detect 85% of major usability problems during conceptual user testing—we anticipated needing five participants to achieve our objective [39]. We obtained written informed consent and offered $120 to participate in the seven-day study.

#### Data collection

Participants were assigned meal-plan calorie levels based on their weight and instructed to follow the plan as written for seven days, with two exceptions: (1) modifying cooking to improve palatability (e.g., adjusting seasonings, cooking times) and (2) substituting ingredients due to unavailability, allergy or strong dislike. In both cases, they were asked to maintain original portion sizes, using plan guidance.

**Fig. 2:**
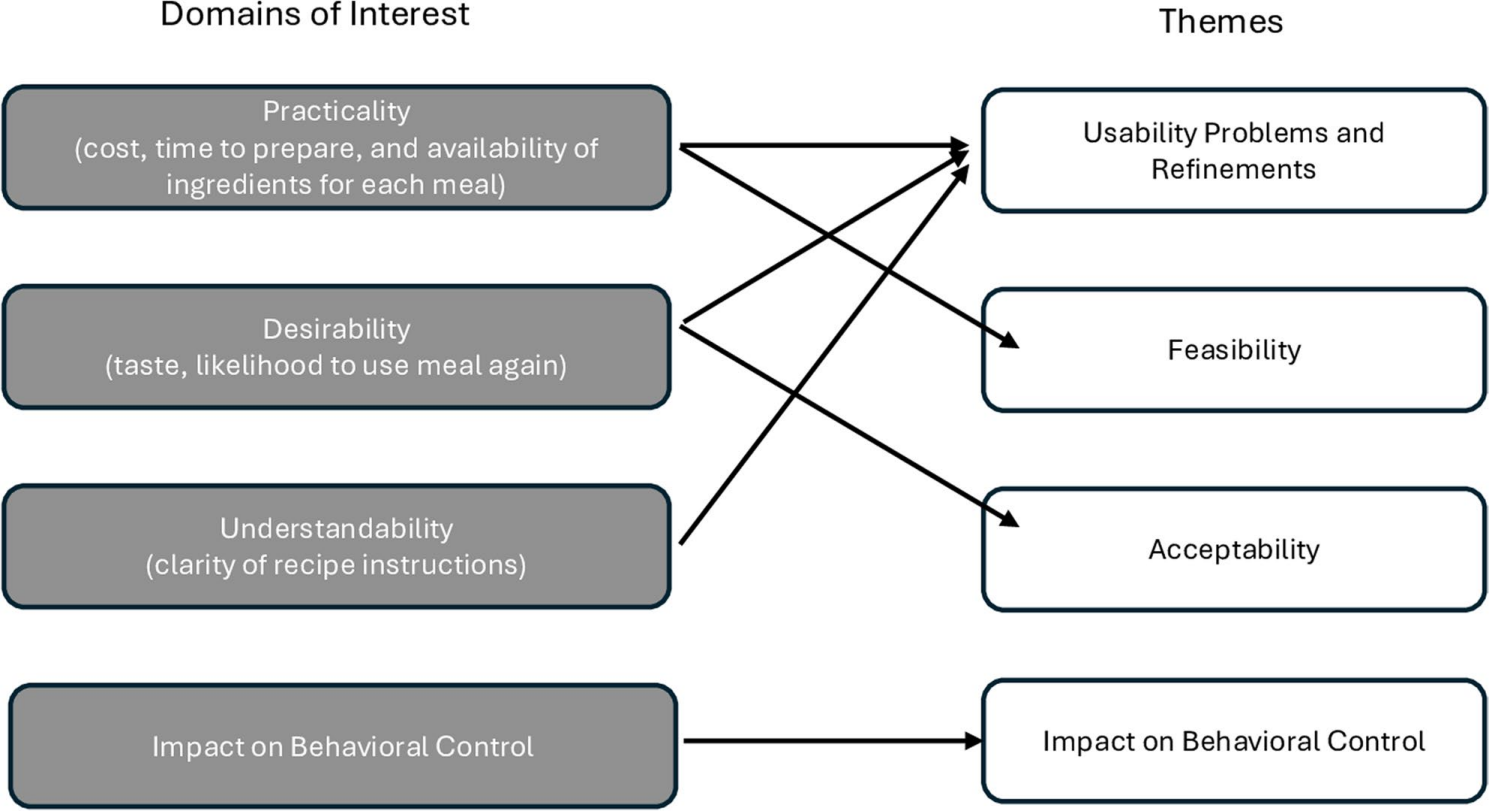
How phase 3’s domains of interest informed themes

We evaluated four domains (Fig. 2): *practicality*, *desirability*, *understandability* and *impact on dietary behavioral control*. Usability problems were assessed based on the first three domains, based on the University of South Carolina’s Drivers of Food Choice framework [40], Vigden’s food literacy model [26], and research on factors influencing meal plan usage [41]. Feasibility and acceptability were assessed using the practicality and desirability domains, respectively.

We used ecological momentary assessment (EMA) surveys and daily semistructured interviews (Appendix B). Each participant was scheduled to complete 21 EMA surveys (3 per day) and 7 interviews. The 11-item EMA survey collected structured feedback about each meal’s cost, preparation time, taste and acceptability in real time [42] and was sent via REDCAP to reach participants three times around their typical reported mealtimes, to obtain immediate feedback [43]. A single text reminder was sent if surveys were not completed within one hour. Study team members and three patient volunteers ( not study participants) reviewed and refined survey items for face validity.

Daily interviews assessed user experiences, followed-up all EMA responses, deeply probed EMA responses indicating low usability or negative reactions and solicited potential improvements. The interview guide covered the three usability domains for meals prepared on the prior day and, on the last day, additionally explored the plan’s impact on eating perceptions and behaviors (Appendix B). Two bilingual female research assistants (IA and PM, both with Bachelor’s degrees in biological sciences) familiar with M/CA cuisines and qualitative interviewing, conducted phone interviews in participants’ preferred language.

#### Data analysis

We used descriptive statistics (counts, proportions) to summarize EMA responses. We performed rapid qualitative analysis of interviews to generate targeted, actionable insights for refining the meal plan, assessing feasibility and acceptability, and exploring its impact on behavioral control [44] .

Interviews were recorded, transcribed, and translated as needed. Using thematic analysis and the matrix method, we organized data using four prespecified domains (practicality, desirability, understandability and behavioral control), with a fifth domain added to document suggestions to fix emerging major meal-plan problems [44] .

For the first two participants, the team (PM, IA, MB) independently reviewed and summarized all 14 interviews, discussing them daily and compiling them into a shared matrix, which they then finalized. Subsequent interviews were summarized using the finalized matrix by one team member and reviewed by the interviewer. Summaries and quotations were organized using Microsoft Excel, with a matrix with rows for participants and columns for domains. The team met several times a week to reconcile discrepancies, flag usability concerns and agree on plan refinements. Data were ultimately grouped into four main themes: meal plan usability problems, feasibility, acceptability and behavioral impact (Fig. 2, Meal Plan Refinements.)

After participants completed the seven-day usability testing, the analysis team and dietitian (ACR) reviewed emergent usability problems to determine whether major, minor, or no changes were needed. *Major problems* necessitated changes to recipe ingredients, preparation steps or written instructions. *Minor problems* could be addressed by how future users would be counseled about the meal plan —either through introductory instructions or verbal guidance. No changes were made in response to feedback based solely on personal preferences. Refinements were made by consensus, incorporating participant input and dietitian expertise; and the updated meal plan was tested by the next user.

## Results

### Phase 1 – meal preparation patterns and preferences

Phase 1 included 19 participants (see Table 1). All were foreign born; most had low acculturation levels. Eleven women and two men were primarily or partially responsible for home meals; the rest relied entirely on a female household member to prepare home meals. Median cost of household groceries was $200 weekly. Interviews lasted a median of 63 min (range 42–86). No new data emerged after interview 16, confirming saturation.

**Table 1:** Participant characteristics

	Phase 1	Phase 3
sample size	19	6
Age (years), median (range)	48 (35–62)	51 (42–73)
Gender (Female) N	12	6
BMI (kg/m^2^)^,^ median (range)	32 (25–62)	37 (26–43)
Medical Conditions*		
MASLD	16	6
Type 2 Diabetes	16	5
Metabolic syndrome	15	4
Birth Country		
Mexico	10	4
El Salvador	7	1
Guatemala	1	1
Honduras	1	0
Acculturation status		
Low (acculturation score ≤ 3)	18	6
High (acculturation score > 3)	1	0
Years in the US, median (range)	26 (10–42)	29 (24–30)
Marital Status		
Married/domestic partner	12	4
Separated/Divorced	4	2
Single/never married	2	0
Widowed	1	0
Household Count, median (range)	4 (2–11)	4 (range 2–7)
Number of children<18yrs in household, median (range)	3 (0–5)	2 (0–3)
Occupation		
Unemployed	0	2
Homemaker	6	1
House Cleaner	4	3
Yard Worker	2	0
Restaurant Worker	2	0
Driver	3	0
Other (white collar job: notary, football agency)	2	0
Food insecurity		
at risk	5	3
not at risk	12	3
Weekly grocery bill, median (range)	$200 ($80-$400)	$160 ($125-$225)
Person responsible for meal preparation at home		
Participant entirely or partially responsible	11	6
Participant’s spouse	3	0
Other member of participant household**	5	0

**Participants’ adult daughter, sister, mother-in-law

Results are reported as number with the characteristic unless otherwise specified.

*Number of participants with the medical condition reported. These are not expected to add up to total number.

Three main themes emerged: source of meals (where meals were prepared or obtained), meal frequency, and meal composition/preferences. Additionally, we inventoried common homemade meal recipes that could be incorporated into the prototype and foods and staples (animal proteins, vegetables, fruits, legumes, grains, cooking oils and appliances) commonly used by participants appropriate for recipe modifications in phase 2 (Supplementary Table 2).

#### Source of meals

Most participants predominantly ate home-cooked meals prepared for the entire household, describing this as part of traditional family life. A few had shifted from regularly eating “outside meals” to “home meals” for health and cost reasons: “When [my health] was okay, and I had my job, I used to eat out at restaurants [often] … but now I don’t … there’s no money and I’m ill” (55 year old man [55yoM]).

Some occasionally ate out – typically on weekends – for a change, to “take a break” from cooking or for social activities like celebrations or church. As a 42-yo mother of two explained, “Usually, everything is from home, but on the weekend [we eat out because] you get tired spending so much time in the kitchen*”.* A few habitually ate weekday lunches, typically fast food, from outside because they found it difficult to prepare or carry a meal to work. “Sometimes I am in places where there is no food. I need to get something fast. . I am a driver, so if it takes one or two hours and then I’m screwed” (48yoM).

A minority predominantly ate outside meals, often alone or with nonhousehold members, typically store or restaurant bought or occasionally from a friend’s home. They did not cook for a variety of reasons “I just get lazy” (56yoF) or “because it’s just for me” (42yoF). Others additionally cited preference and practicality: “I hardly cook at home because I work a lot. . .Also, [outside food] tastes good, it’s cheap, and it’s cooked” (35yoF).

#### Meal frequency/timing

Participants habitually ate two or three meals a day. “Three-meal-a-day” eaters typically ate discrete morning, afternoon and evening meals. A few who woke up late in the day or worked late or night shifts, maintained a “three-meal-a-day pattern” at shifted times, for example, in afternoon, evening, and nighttime. On weekends, this pattern was often disrupted with a preference for two larger meals in late morning/early afternoon and evening, timed around social or family activities.

“Two-meal-a-day” eaters typically skipped breakfast or lunch, eating one “heavy” meal, a second smaller meal, and snacks during the day. For some this was simply a longtime preference: “I usually don’t have breakfast. In the morning … the first meal I eat is lunch” (62yoF). Others were too busy for three meals: “I make only two meals because I don’t have time… we make homemade bread to sell: I get up at 5AM, we have breakfast at 9AM, and sometimes we don’t have time to eat until night when we’re done” (59yoF).

#### Meal composition and preferences

Because the objective of this phase was to characterize homemade meals for adaptation, we focused on homemade meal composition and preferences. Nearly every meal featured an animal-based protein as its primary ingredient: most frequently, eggs for breakfast and chicken or beef for lunch and dinner. Ground turkey, ham deli meats as part of sandwiches, seafood and fish featured among some participants’ meals, but less frequently.

Legumes – most commonly pinto, red, or black beans – often accompanied meals as sides. Non-starchy vegetables (e.g., broccoli, cabbage, squash) were either absent or included in small portions as a side or incorporated into an animal protein-based dish. The most frequently consumed grains were rice, tortillas and bread. Cheese and cream were frequent dairy-based condiments, added for taste.

Participants favored quick, simple meals: “We cook the most regular or simple meals here at the house. . caldo, picadillo, pozole, tamales, enchiladas. We do all those things easily and quickly” (59yoF). They favored chicken- or turkey-based lunches and dinners, as they were quicker to prepare than beef- and pork-based. “Meat is difficult because it takes time. It takes like four hours. I do what’s faster, like turkey or chicken sausages” (42yoF).

Lunch and dinner were the most difficult to prepare. For some, lunch was difficult because of challenges preparing and then carrying food to work. Several mothers felt simultaneously juggling meal preparation with childcare was challenging. “Dinner is difficult … my children arrive from school hungry and want a heavy meal that makes them feel full. So it’s always difficult for me to think what I can prepare for dinner and is also healthy for me” (48yoF).

Generally, participants described liking fruit, vegetables, fish and seafood. Cost was mainly a barrier to purchasing seafood and fruit, as one 35-yo F observed: “I love grilled shrimp … rice, asparagus, salmon. I love baked salmon fillet with asparagus and zucchini. I prepare it, but the main thing that stops me is the price. For a fillet of salmon, it’s $25; and for a package of wieners, you pay $1.50, so you grab the wieners.”

### Phase 2 – meal plan prototype

#### Meal plan structure and options

Three main outputs from phase 1 influenced phase 2’s meal plan prototype construction: meal frequency/timing, meal composition and recipes, and common household staples reported by the population.

Because Phase 1 participants described eating either two (one light and one heavy) or three meals a day, we designed the meal plan to include three meals a day (breakfast, lunch and dinner) of roughly equivalent calorie compositions and one low-calorie snack. This allowed “two-meal-per-day” eaters to eat double the portion of one meal and a single portion of a second meal, while maintaining their prescribed calorie count.

Because Phase 1 participants consistently described meals centered around an animal-based protein, we created a meal plan prototype anchored by a primary animal for each of the 21 meals offered (3 meals a day). We then identified commonly reported Phase 1 recipes that used – or could be adapted to use – these protein anchors. Phase 1 information regarding meal composition, preferences, and typical household availability informed protein selection. Eggs were incorporated into several breakfast items and chicken and turkey into several lunch and dinner items. Though included, we limited meals that that incorporated seafood (due to cost concerns in phase 1) and beef (due to time concerns expressed in phase 1). The prototype included two entirely plant-based meals and three meals (two sandwiches and one taco) that could be quickly prepared and carried to work. We created seven snacks (one per day) using protein sources (cheese, nuts, peanut butter) and fresh fruits reported in Phase 1. Recipes commonly reported in Phase 1 were then mapped to the selected protein anchors and adapted to meet nutritional targets, using commonly available household staples reported in Phase 1.

#### Recipe modifications

Recipe modifications were geared toward achieving the three planned calorie levels (1200, 1500, and 1800 calories/day) and nutrient composition. The most frequently required modifications were reducing fat, changing carbohydrate composition, and increasing portions of high-fiber vegetables in the original recipes. Fat content was reduced by lowering the amount of meat, cheese and avocado in the original recipe and by switching cooking methods from frying to sautéing, baking, or boiling. Complex carbohydrate sources of original recipes – such as rice, corn, beans and tortillas – were retained but in smaller portions. Simple carbohydrates, such as foods and drinks with added sugars (e.g., pan de dulce, sweet teas) were eliminated. Portions of high-fiber vegetables commonly reported in phase 1 (e.g., carrots, squash, green beans, peppers and broccoli) were increased with instructions that they fill half the plate or approximately one cup per meal. Except for dairy- and/or fat-based condiments (e.g., creams, cheese, avocado), original recipes’ condiments and flavorings were retained. One example of recipe modification is traditional chicken flautas. In the original recipe, chicken is wrapped in a tortilla and fried; in the modified version, the amount of meat is reduced, the dish is baked instead of fried, one cup of vegetables as a salad is added, and the serving size of flautas portioned to limit calories consumed.

### Phase 3 – meal plan user testing

Phase 3’s user testing population included six women, median age 51y and BMI 35 kg/m2 (Table 1). All were foreign-born with low acculturation levels, and the median household grocery cost was $160 weekly. Three were at risk for food insecurity.

Based on their weight, three were assigned and tested the 1,800-calorie/day meal plan, two the 1500-calorie/day meal plan, and one the 1200-calorie/day plan. Of 21 meals in the plan, participants collectively prepared 119 meals: three prepared all 21, two prepared 20, and one prepared 16. The EMA response rate was 89% (Supplementary Table 2). Each participant completed seven daily interviews (median 20 min, range 9–41 min). Findings are presented in four sections: user problems and refinements, feasibility, acceptability and impact on behavioral control (Fig. 2). No major usability problems requiring refinements emerged after participant 3, confirming saturation.

#### Usability problems and prototype changes

Major problems with the meal plan primarily related to understandability and desirability. These were identified during interviews with participants 1–3, addressed iteratively with their feedback, and did not emerge in subsequent interviews (Supplementary Table 3). Major understandability problems included a confusing meal plan layout, recipe names not making sense, and a few confusing cooking instructions. In response, we reorganized the plan’s layout, changed recipe titles and revised cooking instructions.

There were two types of desirability problems. The first was a need to substitute the animal protein, complex carbohydrate, or legume called for by a recipe – because of intolerance (e.g., seafood allergy), taste preference (e.g., replacing turkey or chicken with beef or rice with pasta), or familiarity (e.g., replacing red beans with black or pinto beans). Secondly, in a few instances, recipe instructions did not produce a palatable meal. In response, we developed user instructions on how to make isocaloric protein, complex carbohydrates, legume substitutions and improved recipes, using participant feedback.

Two minor problems emerged regarding desirability. First, participants suggested or made meal modifications that would significantly increase fat and calorie content, such as adding avocado, cream, or substituting oatmeal flour for corn flour. In response, we noted the need to emphasize, during both verbal and written counseling, that such modifications can significantly alter the intended calorie content. Secondly, 1200- and 1500-calorie/day plan users felt some meals were too light or left them hungry. In response, we reinforced counseling in the prototype guiding users to add nonstarchy vegetables to meals as a low-calorie strategy to increase meal volume and satiety.

#### Feasibility

Participants found meals’ costs, preparation times, and ingredient availability feasible. They estimated meals cost $2 to $30, with “agreed” or “strongly agreed” across 98% of EMA responses regarding cost acceptability. Qualitative probing of less positive responses (Supplementary Table 2) revealed that they still found costs acceptable, attributing higher expenses to meat, seafood purchases and preparation for multiple family members.

Meal preparation times ranged 5 to 30 min for breakfast, 10 to 120 min for lunches, and 5 to 120 min for dinners. Most found these acceptable, noting that cooking animal proteins (especially chicken and beef), preparing legumes and rice, and measuring ingredients were the most time-consuming tasks.

Participants typically had all necessary ingredients. When unavailable, they substituted or omitted them without difficulty. Several substituted available cheeses when specific cheeses were not available. Commonly missing was turkey, which two participants substituted with another animal protein, and one purchased for testing and reported liking. Other less commonly available ingredients included mushrooms, sesame oil, mustard and balsamic vinegar. A few did not have spicy condiments such as jalapeños, serrano peppers, black pepper, and pepper flakes because of personal preferences. One 67yoF observed: “These recipes you’re providing use what we [Hispanics] all have at home, especially when it comes to the meat. What I don’t usually have is turkey but always have chicken, beef, milanesa, and all the vegetables.”

#### Acceptability

EMA responses across most meals indicated participants agreed or strongly agreed that meals tasted good (98%) and they would reuse the recipe (95%, Supplementary Table 2). Participants universally felt the meals were familiar and acceptable to their families. “Well, this is how I cook. I mean, you’re not giving me recipes that I don’t normally make for myself and my kids. They can also eat this” (49yoF). They also liked exposure to new flavors: “The recipes I’m seeing are something I’m learning from, too; and I’m experimenting with flavors” (42yoF).

Qualitative probing of five “neutral,” “disagree,” or “strongly disagree” responses to reuse the recipe revealed major problems addressed in the refinements. It also uncovered, mainly among lower-calorie-meal-plan (1200 and 1500 calories/day) users, challenges adjusting to the higher vegetable and lower animal protein portions and hunger after some meals. One participant observed: “It was difficult to follow this meal plan. I wasn’t used to it. I would try to stick with it because it has helped me; I feel like I lost weight. … but it was difficult just eating mostly vegetables… and I thought the [portions of protein] was very little.” Participants felt adhering to the meal plan would ultimately require “mentally adjusting, knowing that this is your diet” (49yoF).

#### Impact on behavioral control

Two key subthemes emerged regarding the impact of the meal plan on behavioral control (Supplementary Table 6). First, participants felt the meal plan reframed how they thought about normal portion sizes and came to realize they had previously been overeating: “I’m now seeing the actual amounts that I should eat, I usually cook for five or six people … And you don’t measure portions, you just eyeball it. And, I take portions that are more than I should eat. . now I realize what I actually should be eating” (67yoF).

Secondly, participants felt the plan taught them healthier cooking strategies: how to reduce fat, reduce frying, add vegetables and incorporate lean proteins. They all felt that in addition to providing healthy versions of meals they already typically prepared, the plan taught them how to modify other recipes, outside the plan, using healthier substitutions and methods. It helped them identify leaner animal proteins that would help “reduce fat in my liver,” and how to use them as substitutes for red meats or prepare them if never previously used, like turkey, that they previously may not have been exposed to. Another felt “[with this recipe] I can eat less red meat and make a chicken picadillo. . it’s better to eat chicken and fish.”

## Discussion

While the importance of culturally tailoring dietary interventions is widely recognized, practical guidance on how to do so is limited, particularly for group-based implementation. Our objective was to translate principles of dietetic practice and user testing into a structured, practical approach for developing culturally tailored meal plans for defined patient populations. In the context of a growing burden of obesity-related diseases and a constrained dietetics workforce, there is a pressing need for approaches that extend dietetic principles beyond individualized counseling, are usable by non-dietitian clinicians, and concentrate dietitian expertise where most needed.

### The process

A strength of our design was integration of participant input at every stage, ensuring the intervention was grounded in the experiences and needs of the priority population. Guided by established food choice frameworks and prior research [26, 40, 41], the process addressed key domains of food-related behaviors and preferences. Dietary assessment, contextual adaptation of nutrition guidance, and iterative patient feedback are foundational elements of high-quality dietetic care. However, these practices are typically implemented at the individual level during one-on-one counseling. We organized these elements into a structured framework to support group-based intervention design.

To support replicability, we summarize the framework (Table 2) and corresponding decision rules (Table 3) derived from our implementation experience. Table 2 defines the stepwise process across phases (inputs, key steps, outputs, and suggested sample sizes) and specifies how responsibilities may be distributed across clinical teams. Phases 1 and 3 could be conducted by non-dietitian clinicians or researchers, reserving dietitian expertise for Phase 2 where it is most needed.

**Table 2:** Summarized framework for meal plan development

Phase	Objective	Personnel who leads phase	Inputs	Sample Size*	Key Steps & Methods	Outputs
Phase 1: Population Dietary Characterization	Determine routine meal patterns, practices, and preferences	Non-dietitian clinician or researcher	Diet recall OR diet survey with follow-up interviews	~ 15–20 participants from target population for whom the meal plan is being developed	Assess 3 key domains: 1. meal frequency and timing 2. commonly prepared meals/recipes and common barriers to meal preparation (e.g., cost, time, access) 3. Commonly available household staples *Suggested methods: survey-based assessment with follow-up interviews to clarify responses*	• Meal frequency, timing, and constraints • List of common meals and recipes • List of household staples
Phase 2: Meal Plan Prototype Development	Translate population meal patterns into a nutritionally appropriate, culturally tailored, structured meal plan	Dietitian or trained nutrition professional	1. Phase 1 outputs 2. Nutrition analysis software 3. Clinical nutrition guidelines	Not participant-based	1. Create meal plan skeleton - Map number of meals per day based on Phase 1 patterns - Define calorie composition of each meal - Anchor meals using primary ingredient of Phase 1’s commonly reported meals 2. Map recipes (from Phase 1) onto meal plan skeleton 3. Systematically adapt recipes - Align with nutritional guidelines (e.g., Macronutrient and calorie composition) - Use commonly reported staples for adaptations	• 7-day structured meal plan prototype with standardized recipes and portion guidance
Phase 3: Meal Plan Usability Testing & Refinement	Evaluate and iteratively refine feasibility, acceptability, and usability of the meal plan	Non-dietitian clinician or researcher	1. Meal plan prototype 2. User survey delivered via EMA 3. Follow-interviews interrogating survey items with low Likert ratings	~ 5 participants from target population for whom the meal plan is being developed	1. Assess usability of each meal immediately after consumed 2. Evaluate meals across three predefined usability domains: understandability, desirability, and practicality 3. Iteratively refine meal plan based on user feedback *Suggested methods*: *ecological momentary assessment (post-meal surveys) with follow-up interviews to investigate low ratings (Likert responses < = 3) and guide refinement*	• Identified usability problems • Refined meal plan that resolves major usability problems.

*Suggested sample sizes are based on the current study and intended to support efficient identification of common dietary patterns (Phase 1) and major usability issues (Phase 3).

**Table 3:** Decision rules for meal plan development

Domain	Decision Rule
Phase 2: Meal Plan Prototype Development	
Meal frequency	Design 3 meals/day with flexible portioning to accommodate variation in population-reported eating frequency
Meal structure	Structure meals to provide approximately equivalent caloric content to allow flexibility across eating patterns
Meal options	Anchor meal options using primary ingredients identified from commonly reported Phase 1 meals (e.g., use animal proteins as anchors when they represent dominant components of population-reported meals)
Recipe selection	Select recipes from commonly reported meals identified in Phase 1
Recipe adaptations	Adapt selected recipes to align with nutritional guidelines while preserving cultural relevance
Ingredient selection for recipe adaptations	Prioritize ingredients reported as commonly available staples across participants’ households
Cost constraints	Limit use of ingredients identified as expensive by participants (e.g., seafood)
Time constraints	Limit meals requiring extended preparation time when identified as a barrier in Phase 1
*Phase 3: Meal Plan User Testing and Refinement*	
Usability domains	Evaluate meals across three pre-defined usability domains: understandability, desirability (taste/satisfaction), and practicality (cost, time, ingredient access)
Usability refinement trigger	If EMA survey rating falls below a predefined threshold (e.g. ≤3 on Likert scale) → probe via follow-up interview. If a major usability problem* is identified, implement changes and evaluate the revised meal plan with the next user.*
Meal plan iteration stopping rule	Continue user testing and refinement until no major usability problems are identified across participants (i.e., no new major problems identified in two consecutive users)

*Major usability problem is defined as user feedback requiring modification of recipes or written instructions to improve understandability, desirability, or practicality of the meal plan

In our initial iteration of the process, we used detailed surveys and interviews to identify the information necessary for meal plan development. Phase 1’s interviews provided insight into the priority population’s eating patterns; the findings align with qualitative studies among similar populations, supporting validity of the approach [45, 46]. These data identified three specific aspects of eating behavior central to group-based meal construction: meal frequency/timing, meal composition/preferences, and typical household staples, which serve as key outputs from Phase 1. Phase 3 used EMAs to capture participants’ reactions to meals in their natural environments while minimizing recall bias through in-the-moment responses regarding key food choice domains: taste, convenience, and satisfaction [47, 48]. To our knowledge, our study is the first application of EMAs to evaluate individual meals within a structured meal plan and translation of key usability domains grounded in food literacy and food choice frameworks into survey items to assess the meals. Follow-up qualitative interviews helped contextualize EMA responses and refine the meal plan prototype. Early user testing findings suggest that the participatory design process effectively incorporated the population’s preferences. The data also illustrate how the three predetermined usability domains (understandability, practicality, and desirability) influence usability and guide refinements.

However, the design process took 18 months. To improve efficiency, Phase 1 could use brief surveys to assess the three main domains, reserving interviews for clarification; Phase 3 could rely on EMA surveys to capture key usability metrics, with interviews focused on clarifying negative responses and resolving usability challenges.

To assess its broader applicability, the framework should be evaluated in diverse patient populations. In addition, its application requires implementation within populations that share common sociocultural dietary patterns.

### Name of the institutional the meal plan

The meal plan represents the tangible output of the process. Early conceptual testing suggests the process produced a feasible, acceptable, and usable meal plan for M/CA patients with MASLD. Qualitative assessment suggests that it may enhance key aspects of behavioral control, such as recognizing appropriate portion sizes and improving dietary quality of meals. This aligns with prior research showing that structured meal plans – through meal planning and specific portion size guidance –reduce the cognitive load associated with dietary decisions and thereby, facilitate dietary adherence [22, 49].

The meal plan was tailored for M/CA patients who prepare traditional home-cooked meals and, thus, may be less applicable for more acculturated groups or those less willing to cook. However, the design process presented here could be applied to design appropriately meal plans for other populations. Larger-scale testing –currently ongoing in a pilot feasibility trial among 50 participants [50] **–** is necessary to determine real-world meal plan usage and dietary adherence and may uncover additional usability problems. Effectiveness trials are required to assess the meal plan’s long-term impact on behavioral control and dietary behaviors.

## Conclusions

This study presents a patient-centered framework for developing culturally tailored, group-based meal plans. By integrating behavioral theory, qualitative methods, and user testing, it provides a practical approach for developing culturally aligned dietary interventions in settings with limited dietitian access. The resulting meal plan is an example of how this approach can generate feasible, acceptable tools to support dietary behavioral control among M/CA patients with MASLD, and may inform intervention development for other populations.

## Supplementary Information

Supplementary Material 1.

Supplementary Material 2.

Supplementary Material 3.

Supplementary Material 4.

Supplementary Material 5.

Supplementary Material 6.

Supplementary Material 7.

Supplementary Material 8.

Supplementary Material 9.

## Data Availability

The full individual‐level survey responses, qualitative transcripts, and audio recordings from this study contain sensitive participant information, and therefore cannot be made publicly available in order to protect confidentiality. Aggregated data—including survey responses by question, thematic summaries, and anonymized exemplar quotations—are provided in the Supplementary Materials. De-identified summary tables beyond what is included may be shared by the corresponding author upon reasonable request, subject to approval by the ethics committee and/or institutional review board.

## Abbreviations

BMI: Body Mass Index
EMA: Ecological Momentary Assessment
F: Female
M: Male
MASLD: Metabolic-dysfunction associated steatotic liver disease
M/CA: Mexican and Central American
Y: Years
P: Participant
