# Supplementary material for: Applying user-centered design to develop a culturally sensitive, low-calorie meal plan for enhancing dietary behavioral control in MASLD
Source: BMC Nutr. 2026 May 6;12:123. doi: 10.1186/s40795-026-01347-8 (PMC13312602; doi:10.1186/s40795-026-01347-8)
Supplement: Supplementary file 1 — Supplementary Material 1. [file 40795_2026_1347_MOESM1_ESM.docx]

| **Supplementary Table 1. Foods, Staples, and Cooking Appliances Commonly Used by Phase 1 Participants** | |
| --- | --- |
| **Item** | **Number of participants reporting items as typically at home**  **(total participants = 19)** |
| **Protein** | |
| Chicken | **16** |
| Fish | **14** |
| Beef | **11** |
| Turkey | **7** |
| Pork | **6** |
| Deli Meat (ham) |  |
| *Items reported by fewer than 5 participants: shrimp, octopus, ham deli meat* | |
| **Vegetables** | |
| Carrot | **14** |
| Squash (including chayote and zucchini) | **13** |
| Onion | **9** |
| Lettuce | **8** |
| Tomato | **7** |
| Cabbage | **5** |
| Broccoli | **5** |
| *Items reported by fewer than 5 participants: Avocado, Celery, Cilantro, Corn, Cucumber, Garlic, Green beans, Beets, Onion, Peppers, Potato, Spinach, Cactus* | |
| **Fruits** | |
| Banana | **18** |
| Apples | **11** |
| Oranges | **7** |
| Strawberries | **7** |
| Tangerines | **6** |
| Watermelon | **6** |
| *Items reported by fewer than 5 participants: Cantaloupe, Grapes, Mango, Blackberries, Blueberries, Peaches, Raspberries, Guavas, Kiwi, Pears, Pineapple, Plums* | |
| **Legumes** | |
| Pinto | **11** |
| Black | **9** |
| Red | **7** |
| Lentils | **5** |
| *Items reported by fewer than 5 participants: Chickpeas, White, Bayo, Peruvian, Salvadorian* | |
| **Grains** | |
| Tortillas | **14** |
| White rice | **13** |
| Wheat bread | **6** |
| *Items reported by fewer than 5 participants: White Bread, Jasmine Rice, Quinoa, Basmati Rice* | |
| **Oil** | |
| Olive | **8** |
| Mazola corn | **5** |
| *Items reported by fewer than 5 participants: Vegetable, Butter, Avocado, Canola, Lard* | |
| **Seasoning** | |
| Salt | **13** |
| Pepper | **10** |
| Garlic salt | **8** |
| Garlic | **6** |
| Cumin | **5** |
| Knorr chicken bouillon | **5** |
| *Items reported by fewer than 5 participants: Onion Powder, Rosemary, Lemon, Oregano, Paprika* | |
| **Appliances** | |
| Microwave | **13** |
| Blender | **13** |
| Elective stove | **9** |
| Oven | **9** |
| Gas stove | **7** |
| Toaster | **5** |
| Air fryer | **5** |
| *Items reported by fewer than 5 participants: Coffeemaker, Pressure Cooker, Juice Extractor, Steamer, Molcajete, Grill* | |
