# Supplementary material for: Applying user-centered design to develop a culturally sensitive, low-calorie meal plan for enhancing dietary behavioral control in MASLD
Source: BMC Nutr. 2026 May 6;12:123. doi: 10.1186/s40795-026-01347-8 (PMC13312602; doi:10.1186/s40795-026-01347-8)
Supplement: Supplementary file 2 — Supplementary Material 2. [file 40795_2026_1347_MOESM2_ESM.docx]

| **Supplementary Table 2. Phase 3 Ecological Momentary Assessment Survey Questions & Responses** | | | | | | | | | | | | | | | | | | | | | | |
| --- | --- | --- | --- | --- | --- | --- | --- | --- | --- | --- | --- | --- | --- | --- | --- | --- | --- | --- | --- | --- | --- | --- |
|  | **Day 1** | | | **Day 2** | | | **Day 3** | | | **Day 4** | | | **Day 5** | | | **Day 6** | | | **Day 7** | | |  |
|  | **Breakfast** | **Lunch** | **Dinner** | **Breakfast** | **Lunch** | **Dinner** | **Breakfast** | **Lunch** | **Dinner** | **Breakfast** | **Lunch** | **Dinner** | **Breakfast** | **Lunch** | **Dinner** | **Breakfast** | **Lunch** | **Dinner** | **Breakfast** | **Lunch** | **Dinner** |  |
| **Meal Name** | **Huevo ala Mexicana (Mexican eggs)** | **Modified pollo milanesa (Flattened chicken)** | **Caldo de pollo (Chicken soup)** | **Desayuno de espinacas y huevo (Spinach and egg)** | **Salteado de verduras mixtas y arroz (Mixed vegetables stir-fry and rice)** | **Bistec de res con salsa chimichurri (Beef steak with chimichurri Sauce)** | **Atole de avena (Oatmeal atole)** | **Taco de frijoles (Bean taco)** | **Salmon y brocoli (Salmon and broccoli)** | **Desayuno tostada de aguacate (Breakfast avocado toast)** | **Picadillo de pavo con tostada (Turkey picadillo con tostada)** | **Ensalada de verduras con pollo y vinagreta (Vegetable salad with chicken and vinaigrette)** | **Sandwich de pavo y aguacate (Turkey-avocado sandwich)** | **Tazon de burrito (Beef burrito bowl)** | **Sopa de lentejas (Lentil soup)** | **Berrillivious-waffle de platano (Berrilicious-banana waffle)** | **Flautas de pollo al horno (Baked chicken flautas)** | **Sandwich clasico de ensalada de pollo (Classic chicken salad sandwich)** | **Chilaquiles verdes (Chilaquiles verdes)** | **Mariscada sencilla (Simple mariscada)** | **Chile relleno de pollo (Chicken stuffed chile relleno)** |  |
| **Event Name** | **Meal 1** | **Meal 2** | **Meal 3** | **Meal 4** | **Meal 5** | **Meal 6** | **Meal 7** | **Meal 8** | **Meal 9** | **Meal 10** | **Meal 11** | **Meal 12** | **Meal 13** | **Meal 14** | **Meal 15** | **Meal 16** | **Meal 17** | **Meal 18** | **Meal 19** | **Meal 20** | **Meal 21** | **Total response, count (percent)** |
| **Meal Users** |  |  |  |  |  |  |  |  |  |  |  |  |  |  |  |  |  |  |  |  |  |  |
| # who prepared meal | 6 | 6 | 6 | 6 | 5 | 5 | 6 | 6 | 6 | 6 | 5 | 6 | 6 | 6 | 6 | 4 | 6 | 6 | 6 | 4 | 6 | 119 meals prepared |
| Reason meal not prepared (among non-users) |  |  |  |  | no time to prepare | unfamiliar with chimichurri |  |  |  |  | no time to prepare |  |  |  |  | no time to prepare |  |  |  | seafood allergy (1); dislikes seafood (1) |  |  |
| **EMA response**  *(#users who completed EMA corresponding to meal)* | 6 | 4 | 4 | 5 | 4 | 5 | 6 | 6 | 6 | 5 | 5 | 6 | 4 | 6 | 5 | 4 | 5 | 6 | 6 | 4 | 4 | 106 EMAs completed (89%) |
| **EMA survey questions and responses** | | | | | | | | | | | | | | | | | | | | | | |
| ***Question 1. For how many people did you prepare the meal including yourself? (Ex. If you prepared the meal for 3 family members + yourself, you should answer 4)   ¿Para cuántas personas preparo la comida? (Ej. Si preparó la comida para usted + 3 miembros de la familia, debe responder 4)*** | | | | | | | | | | | | | | | | | | | | | | |
| 1 | 4 | 3 | 3 | 4 | 4 | 4 | 6 | 6 | 5 | 6 | 5 | 5 | 5 | 6 | 5 | 4 | 5 | 6 | 6 | 4 | 4 | 96 (96%) |
| 2 | 1 |  |  | 1 |  |  |  |  |  |  |  |  |  |  |  |  |  |  |  |  |  | 2 (2%) |
| 3 |  |  |  |  |  |  |  |  |  |  |  |  |  |  |  |  |  |  |  |  |  | 0 |
| 4 | 1 | 1 |  |  |  | 1 |  |  | 1 |  |  | 1 |  |  |  |  |  |  |  |  |  | 5 (5%) |
| 5+ |  |  |  |  |  |  |  |  |  |  |  |  |  |  |  |  |  |  |  |  |  | 0 |
| *Total responses* |  |  |  |  |  |  |  |  |  |  |  |  |  |  |  |  |  |  |  |  |  | *103* |
| ***Question 2. Which version of the recipe did you use?¿Qué versión de la receta uso?*** | | | | | | | | | | | | | | | | | | | | | | |
| good for 1 serving | 4 | 3 |  | 4 | 4 | 4 | 6 | 6 | 5 | 6 | 5 | 5 | 5 | 6 | 5 | 4 | 5 |  | 6 | 4 | 4 | 87 (93%) |
| good for 4 servings | 1 | 1 |  |  |  | 1 |  |  | 1 |  |  | 1 |  |  |  |  |  |  |  |  |  | 5 (5%) |
| other | 1 |  |  | 1 |  |  |  |  |  |  |  |  |  |  |  |  |  |  |  |  |  | 2 (2%) |
| Total responses |  |  |  |  |  |  |  |  |  |  |  |  |  |  |  |  |  |  |  |  |  | *94* |
| ***Question 3. Did the recipe require you to purchase any ingredients that you do not typically buy or have in your home? ¿La receta requería que comprara algunos ingredientes que normalmente no compra o tiene en su casa?*** | | | | | | | | | | | | | | | | | | | | | | |
| I had everything | 6 | 3 |  | 5 | 4 | 5 | 6 | 5 | 6 | 6 | 4 | 6 | 5 | 6 | 5 | 4 | 5 | 6 | 6 | 2 | 3 | 95 (95%) |
| I had to buy a new ingredient |  | 1 |  |  |  |  |  | 1 |  |  | 1 |  |  |  |  |  |  |  |  | 2 | 1 | 5 (5%) |
| Total responses |  |  |  |  |  |  |  |  |  |  |  |  |  |  |  |  |  |  |  |  |  | *100* |
| ***Question 4. How long did it take to prepare the entire meal? (Think about how long it took you to prepare the meal, from the moment you started preparing the ingredients to cooking completion.) ¿Cuánto tiempo se tardó en preparar toda la comida? (Piensa en cuánto tiempo se llevó en preparar la comida, desde el momento en que comenzó a preparar los ingredientes hasta que terminó de cocinar.)*** | | | | | | | | | | | | | | | | | | | | | | |
| Time reported (minutes) | 9-60 | 15-80 | 5-120 | 5-30 | 15-30 | 15-30 | 8-15 | 10-30 | 10-60 | 5-20 | 20-30 | 15-30 | 5-20 | 15-90 | 15-90 | 12-30 | 10-120 | 8-30 | 8-30 | 12-30 | 10-90 |  |
| ***Question 5. How much do you think the meal cost? (Think about the ingredients and quantities of various ingredients you used to prepare this meal.)   ¿Cuánto crees que cuesta la comida?  (Piense en los ingredientes y las cantidades de estos varios ingredientes que usaste para preparar esta comida.)*** | | | | | | | | | | | | | | | | | | | | | | |
| Cost reported (dollars) | $2-$10 | $8-$20 | $5-$30 | $2-$20 | $2-$20 | $5-$20 | $2-$20 | $2-$25 | $3-$25 | $2-$15 | $2-$20 | $3-$15 | $1.50-$15 | $2-$20 | $2-$15 | $2-$14 | $3-$15 | $2-$15 | $2-$10 | $5-$20 | $3-$15 |  |
| ***Question 6. The cost of the meal was acceptable.   El costo de la comida fue acceptable.*** | | | | | | | | | | | | | | | | | | | | | | |
| strongly disagree, disagree, or neutral  (Likert response 1 through 3) | 0 | 0 | 0 | 0 | 0 | 1 | 0 | 0 | 0 | 0 | 0 | 0 | 0 | 0 | 0 | 1 | 0 | 0 | 0 | 0 | 0 | 2 (2%) |
| agree or strongly agree  (Likert response 4 through 5) | 6 | 4 | 0 | 5 | 4 | 4 | 6 | 6 | 6 | 6 | 5 | 6 | 5 | 6 | 5 | 3 | 5 | 6 | 6 | 4 | 4 | 98 (98%) |
| Total responses |  |  |  |  |  |  |  |  |  |  |  |  |  |  |  |  |  |  |  |  |  | *100* |
| ***Question 7. The meal tasted good.   La comida sabía bien.*** | | | | | | | | | | | | | | | | | | | | | | |
| strongly disagree, disagree, or neutral  (Likert response 1 through 3) | 0 | 0 | 0 | 0 | 0 | 1 | 0 | 0 | 0 | 0 | 1 | 0 | 0 | 0 | 0 | 0 | 0 | 0 | 0 | 0 | 0 | 2 (2%) |
| agree or strongly agree  (Likert response 4 through 5) | 6 | 4 | 0 | 5 | 4 | 4 | 6 | 6 | 6 | 6 | 4 | 6 | 5 | 6 | 5 | 4 | 5 | 6 | 6 | 4 | 4 | 98 (98%) |
| Total responses |  |  |  |  |  |  |  |  |  |  |  |  |  |  |  |  |  |  |  |  |  | *100* |
| ***Question 8. I would use this recipe often.   Usaría esta receta frecuentemente.*** | | | | | | | | | | | | | | | | | | | | | | |
| strongly disagree, disagree, or neutral  (Likert response 1 through 3) | 0 | 0 | 0 | 0 | 0 | 0 | 1 | 0 | 0 | 0 | 1 | 0 | 0 | 1 | 1 | 0 | 0 | 0 | 0 | 1 | 1 | 5 (5%) |
| agree or strongly agree  (Likert response 4 through 5) | 6 | 4 | 0 | 5 | 4 | 5 | 5 | 6 | 6 | 6 | 4 | 6 | 5 | 5 | 4 | 4 | 5 | 6 | 6 | 3 | 3 | 95 (95%) |
| Total responses |  |  |  |  |  |  |  |  |  |  |  |  |  |  |  |  |  |  |  |  |  | *100* |
| ***Question 9. Did you make any changes to the recipe?   ¿Hizo algún cambio en la receta?*** | | | | | | | | | | | | | | | | | | | | | | |
| No | 5 | 1 |  | 4 | 4 | 5 | 4 | 6 | 5 | 5 | 4 | 6 | 3 | 6 | 4 | 3 | 5 | 6 | 6 | 2 | 4 | 84 (85%) |
| Yes | 1 | 3 |  | 1 |  | 0 | 2 |  | 1 | 1 | 1 |  | 2 |  | 1 | 1 |  |  |  | 1 |  | 15 (15%) |
| Total Responses |  |  |  |  |  |  |  |  |  |  |  |  |  |  |  |  |  |  |  |  |  | *99* |
| ***Question 10 . Did the recipe work as written? ¿Funcionó la receta como está escrita?*** | | | | | | | | | | | | | | | | | | | | | | |
| No |  | 1 |  |  |  | 0 | 1 | 1 | 1 | 1 |  |  | 1 |  | 1 |  |  | 1 |  |  |  | 8 (8%) |
| Yes | 6 | 3 |  | 5 | 4 | 5 | 5 | 5 | 5 | 5 | 5 | 6 | 4 | 6 | 4 | 4 | 5 | 5 | 6 | 3 | 4 | 91 (92%) |
| Total responses |  |  |  |  |  |  |  |  |  |  |  |  |  |  |  |  |  |  |  |  |  | *99* |
| ***Question 11. The recipe was easy to use.   La receta era fácil de usar.*** | | | | | | | | | | | | | | | | | | | | | | |
| strongly disagree, disagree, or neutral  (Likert response 1 through 3) | 0 | 0 | 0 | 0 | 0 | 0 | 0 | 0 | 0 | 0 | 0 | 0 | 0 | 0 | 1 | 0 | 1 | 0 | 0 | 1 | 0 | 3 (3%) |
| agree or strongly agree  (Likert response 4 through 5) | 6 | 4 | 0 | 5 | 4 | 5 | 6 | 6 | 6 | 5 | 5 | 6 | 5 | 6 | 4 | 4 | 4 | 6 | 6 | 3 | 4 | 100 (94%) |
| Total responses |  |  |  |  |  |  |  |  |  |  |  |  |  |  |  |  |  |  |  |  |  | *103* |
| *Questions 5 through 10 allowed for Likert responses with scale as presented.* | | | | | | | | | | | | | | | | | | | | | | |
