# Supplementary material for: Applying user-centered design to develop a culturally sensitive, low-calorie meal plan for enhancing dietary behavioral control in MASLD
Source: BMC Nutr. 2026 May 6;12:123. doi: 10.1186/s40795-026-01347-8 (PMC13312602; doi:10.1186/s40795-026-01347-8)
Supplement: Supplementary file 3 — Supplementary Material 3. [file 40795_2026_1347_MOESM3_ESM.docx]

| **Supplementary Table 3. Phase 3 User Feedback and Corresponding Meal Plan Refinements** | | | | | | | |
| --- | --- | --- | --- | --- | --- | --- | --- |
| **Participant** | **Demographics** | **Meal plan tested** | **Major Problems** | **Major Changes Made** | **Minor Problems** | **Minor Changes Made** | **Personal Preferences Feedback  (not requiring changes)** |
| 1 | 46F,  House cleaner,  El Salvadorian | 1800 cal/day | UNDERSTANDABILITY:  -Layout of recipes and pictures confusing  DESIRABILITY:  -Did not prepare seafood meals because of allergies -substituted beef for chicken across a few recipes based on preference -Made carbohydrate substitutions: rice with pasta (M3) | 1. Reorganized layout per participant suggestions  2. Created protein substitution section in the introduction section to allow isocaloric exchanges across animal, dairy, and plant based proteins  3. Created carbohydrate substitution section to guide isocaloric exchanges | DESIRABILITY:  -Added cream (M12, notable because adds fat/calories) | Noted that counseling should point out to users that adding sources of fat will increase calorie content of meal | DESIRABILITY:  M3: Felt soup was too light for dinner and more appropriate for a lunch. M6: Did not prepare because unfamiliar with chimichurri. Eliminated jalepenos and other spicy condiments and ingredients because of heartburn Altered seasonings to taste  PRACTICALITY:  M2: Prepared fresh salad instead of cooked vegetables because it was faster |
| 2 | 42F,  House cleaner, Guatemalan | 1800 cal/day | UNDERSTANDABILITY:  -M8, 14, 21: Original recipe title does not make sense  - M10, 15: Recipe instructions do not work as written -Instructions about portions or cuts of animal proteins to be used across various recipes are confusing and not aligned with how user buys meat from store.  DESIRABILITY:  - M7, M10,M13, M15: Original recipe didn't work; prepared it twice and suggested changes, based on second preparation. -M14: Offered that lettuce could be used as a wrap in place of tortilla -Prefers red beans | 1. Changed name of M8 recipe  2. Changed name of M14 and 21 after P3 noted a similar problem  3. Created a section in introduction discussing cuts of meat and clarified portion sizes through the meal plan's recipes.  4. M3, 7, 10, 13, 15 - Altered the recipe per suggestions 6. Created legume substititon section in the introduction. | DESIRABILITY:  -Expressed ground meat as preferred animal protein across several dinner and lunch items (e.g./ M11) -Suggested greater portion of animal protein (for example sausage to M15), added an additional egg to M4, added avocado to M2: notable suggestions because these changes add calories.  PRACTICALITY:  -Wherever instructions were to bake an item she used air fryer. | Noted that counseling should point out to users that increasing animal protein, sources of fat or adding them to meals that don't have them will change the calorie composition of the meal.  Added air frying (along with baking) as an alternative to frying | DESIRABILITY:  M4, M6: Felt these were too heavy.  M20: Felt it needed more vegetables; altered seasonings to taste. M2: Removed carrots from M2 Made nonstarchy vegetable substitutions based on preference  PRACTICALITY:  M2: Grilled instead of sauteing because faster |
| 3 | 67F, Homemaker, Mexican | 1800 cal/day | UNDERSTANDABILITY:  -M14, 20, 21: Original recipe title doesnt work  - M1, 2, 13, 14: Written instructions were confusing  DESIRABILITY:  -M1, 7,10, 13, 15 - Recipe didn't work. | 1. Changed name of recipes (M14,21)  2. Changed recipe instructions and ingredients according to her suggestions (M1,2,7,10, 13,14,15) | DESIRABILITY:  -M16: Substituted corn flour with oat flour: notable because higher calorie density | Noted to counsel users to be aware of potential of how substituting flours can change calories of a meal | DESIRABILITY:  Used tilapia instead of cod; substituted chicken for turkey (which she doesn't typically buy) Substituted type of cheese, but used same portion size  PRACTICALITY:  Substituted red beans with pinto beans |
| 4 | 52F, Homemaker, Mexican | 1500 cal/day | none | none | none | none | DESIRABILITY:  Avoided "spicy" ingredients  PRACTICALITY:  Made isocalorie substitutions for ingredients she didn't have (for, e.g., red beans) or eliminated them altogether (vinegar, mustard, mushrooms) |
| 5 | 49F,  Custodian, Mexican | 1200 cal/day | none | none | DESIRABILITY:  Felt 3 meals were too light | Noted to reinforce information already in the meal plan instructing users that they can add nonstarchy vegetables as a low-calorie way to increase volume of food and satiety | DESIRABILITY:  Avoided spicy ingredients (black pepper, flakes)  Substituted turkey (doesn't like it) with equivalent portion beef  Substituted cheeses with what she had  Used egg whites in place of eggs with yolk |
| 6 | 52F, Homemaker, Mexican | 1500 cal/day | none | none | DESIRABILITY:  Felt hungry after 2 meals | none | PRACTICALITY:  Substituted red beans with black  DESIRABILITY Substituted fish (doesn’t like it) with chicken M4,5,11,15: Felt they they needed higher portion of animal protein, addition of bacon and smaller portion of vegetables |
