# Supplementary material for: Applying user-centered design to develop a culturally sensitive, low-calorie meal plan for enhancing dietary behavioral control in MASLD
Source: BMC Nutr. 2026 May 6;12:123. doi: 10.1186/s40795-026-01347-8 (PMC13312602; doi:10.1186/s40795-026-01347-8)
Supplement: Supplementary file 4 — Supplementary Material 4. [file 40795_2026_1347_MOESM4_ESM.docx]

| **Supplementary Table 4.Phase 3 Meal Plan Feasibility: Sample Quotes Reflecting Perceptions of Meal Plan's Practicality Related to Cost, Time, and Ingredient Availability** |  |
| --- | --- |
| ***Meal costs and preparation times were acceptable and used ingredients typically at home.*** | |
| [I would use this recipe again because] Its something easy and uses what you have on hand all the time | P2 (about D3 lunch) |
| It’s very easy and it’s economical as well. If you have leftover beans, you can make a salad or a mix with that. Usually, you have rice at home. So it’s something that you always have at home. | P2 (about D5 lunch) |
| Everything was cheap, all the vegetables, and lentils are very economical . . . and the girls like it a lot and, well, I also like it a lot. | P3 (about D5 dinner) |
| No, actually I have everything at home. These recipes you’re providing are ... all good [for] us Hispanics. It’s what we all have at home, especially when it comes to the meat, I mean the protein . . .What I don’t usually have is turkey. But you always have chicken, you always have beef, you always have milanesa, and all the vegetables, well, those are what one always has at home. Everything I already buy, right? So, no, I haven’t struggled because I have everything at home. | P3 about the meal plan in general |
| I don’t usually buy or like turkey, but in this sandwich, I did like it | P4 (about D5 breakfast) |
| It's quick and practical. Normally, we cook with more things, and it takes longer. This was very practical because it was just grilled—everything sautéed—and done quickly. | P5 (about D2 dinner) |
| I really like salmon, but I don’t cook it often because there are so many people in the house. It’s hard to make enough portions for everyone. But, once a week, I think I can afford that. | P5 (about D3 dinner) |
| Because it’s easy to make, quick, and it’s good. Plus, it doesn’t make you feel too full in your stomach, it’s just right. | P6 (about D3 lunch) |
| ***Potential feasiblity barrier - Measuring ingredients was the most difficult part of the meal plan*** | |
| No. It wasn’t difficult. It’s simple. It’s just the measurements that need to be done, and that’s what takes time, but it’s not hard. | P5 about the meal plan in general |
| The hardest part is the measurements, but the rest is easy; and the food isn’t too heavy, and you have all the ingredients. | P6 (about D5 lunch) |
