# Supplementary material for: Applying user-centered design to develop a culturally sensitive, low-calorie meal plan for enhancing dietary behavioral control in MASLD
Source: BMC Nutr. 2026 May 6;12:123. doi: 10.1186/s40795-026-01347-8 (PMC13312602; doi:10.1186/s40795-026-01347-8)
Supplement: Supplementary file 5 — Supplementary Material 5. [file 40795_2026_1347_MOESM5_ESM.docx]

| **Supplementary Table 5. Phase 3 Meal Plan Acceptability: Sample Quotes Reflecting Perceptions of Meal Plan's Desirability Related to Taste and Willingness to Prepare Again** | |
| --- | --- |
| ***Meals were familiar and would prepare several of them again*** | |
| Ah, [I would make it again] because it’s good. For example, I’ve used it before. My Guatemalan husband, they do have atole de avena, so I already had some experience making it. | P1 (about D3 breakfast) |
| I'd prepare this again because it had a combination that made all the different ingredients come together a bit more. At lunchtime, it helped with hunger and left the stomach satisfied. | P1 (about D3, Lunch) |
| I really think that when you cook very similarly to those meals, you already know more or less what it is, and you have all the common ingredients ready at home. So for me, it was easy to use the recipe. It’s just about reading the steps because for one... | P2 (about D4 lunch ) |
| These meals that you included here in all the recipes, from the first day to the last, are what I actually cook. It's what is really cooked in the Hispanic kitchen. For example, the omelet, the scrambled eggs with spinach, well that’s an omelet that you usually have for breakfast . . . these are very common meals. | P3 about the meal plan in general |
| Look, all these recipes you provided are what I cook every week. So that’s why I say I’ll do it again, because it’s what I eat … it is very nutritious, and the girls like it a lot and well, I also like it a lot. | P3 (about D2 dinner) |
| it’s a meal I usually make one to two times a week. | P3 (about D1 dinner) |
| Yes, well, this is how I cook. I mean, you're not giving me recipes that I don’t normally make for myself and my kids. They can also eat this. | P5 (about D1 lunch) |
| Ah, yes, yes. I think I’m going to make it a habit, regardless of whether it’s part of the 7-day plan. I think I’ll keep it for myself. | P5 (about D1 breakfast) |
| Because I thought it was delicious and healthy, and I think I’ll make it for my kids too. Well, in fact, all the recipes we’ve been working on will become part of my family’s menu. | P5 (about D5 dinner) |
| I liked it, I really liked the chimichurri. I would definitely recommend this meal. I would make this meal again… I would recommend it to someone else to eat. | P6 (about D2 dinner) |
| ***Liked exposure to new tastes and ingredients*** | |
| The recipes I’m seeing are something I’m learning from, too, and I’m experimenting with flavors. There are some flavors that I usually make, like salads, and sometimes I mix vegetables and even beans, but the taste is different because of the seasonings, like the powdered ones and the vinaigrette that was prepared. It changes the flavor. | P2 about the meal plan in general |
| Lately, I’ve been trying new flavors with your recipes. Normally, with Mexican food, we always use onion, tomato, and chili—always onion, tomato, and chili. So trying new seasonings, little things like the flavor of paprika, the taste of broccoli with the salmon juice—it’s all different. I liked it. | P5 about the meal plan in general |
| Well, maybe the soy sauce gave it a good flavor, I had never used it. So, it tasted different; I’d never had it before. I liked how it tasted with that soy sauce. | P6 (about D1 lunch) |
| ***Potential acceptability barrier - Lower calorie meal plan users described difficulty adjusting to higher vegetable portions, small meat portions, and feeling hungry after some meals.*** | |
| When I ate it, I was fine; but after about three hours, I was hungry again. | P5 (about D2 breakfast) |
| Expressed that she did not immediately like this meal: “As I told you, I need to get used to eating meals like this with so many combinations [lot of ingredients]” Like the zucchini, I didn’t really like the combination with the spinach and the pepper. … it’s just that I’m not used to making that in the morning with so many things.” | P5 (about D2 breakfast) |
| In response to whether she had anything else good or bad to comment about the meal: “Ah, no, the problem was that my kids brought food from outside, and I was dying to eat a bite; but they said no. It’s just a matter of mentally adjusting, knowing that this is your diet, and this is what you can eat.” | P5 (about D1 lunch) |
| It was difficult to follow this meal plan. I wasn’t used to it. I would try to stick with it because it has helped me, I feel like I lost weight. I haven’t weighed myself yet, but I feel like my clothes fit a little looser, and, yes, it has helped me. Interviewer: What aspects of meal plan were difficult for you? Participant: “Just eating mostly vegetables. Yes, I always complemented it with protein, but chicken. That’s what I found difficult, and I thought it [portions of protein] was very little.” | P6 about the meal plan in general |
