# Supplementary material for: Applying user-centered design to develop a culturally sensitive, low-calorie meal plan for enhancing dietary behavioral control in MASLD
Source: BMC Nutr. 2026 May 6;12:123. doi: 10.1186/s40795-026-01347-8 (PMC13312602; doi:10.1186/s40795-026-01347-8)
Supplement: Supplementary file 6 — Supplementary Material 6. [file 40795_2026_1347_MOESM6_ESM.docx]

| **Supplementary Table 6.Phase 3 Meal Plan Impact on Behavioral Control: Sample Quotes** | Study ID |
| --- | --- |
| **More aware of appropriate portion sizes** | |
| It shows how one should be eating in a suitable way. | P1 (about D2 lunch) |
| [With this meal plan] the difference is that the food is portioned, so it’s not a large quantity like you’re used to serving yourself because you don't measure it. That’s the difference I felt—it’s measured . . . But I felt satisfied. | P2 about the meal plan in general |
| So, I’m now seeing the actual amounts that I should eat, the carbohydrates I really need during the day… I realize what I actually should be eating | P3 about the meal plan in general |
| I like the portion sizes. That was the thing I didn’t know how to do — portion my meals. I would just serve myself and that was it. That’s also why I gained a lot of weight, because I would just serve myself a big plate and that was it. Until I realized with you guys in the book that I had to portion my food for each person. | P5 about the meal plan in general |
| I’ve felt much better with the portions because I don’t feel so bloated in my stomach. I don’t get so full, and I feel like it’s helped me with my sugar. | P5 about the meal plan in general |
| Well, it has changed because before, I would serve myself, like, what, a spoonful, which wasn’t really a measurement, and I would just eat it. I ate the rice and beans together. But here, you eat beans, but it’s not as much beans. It’s more balanced, and that’s what I’ve noticed and picked up. | P6 about the meal plan in general |
| Before, I would serve myself and not really a measure [my food]. I would just eat. But here, you eat beans, but it’s not as much beans. It’s more balanced … that’s what I’ve noticed and picked up . | P6 about the meal plan in general |
| **Learned how to add more vegetables to typical meals** | |
| I always [make this recipe] but without the potato, without the carrot, it's just pure meat and I add the sauce with pasta. But, I mean, I still say that this recipe offers a new way to eat the meal | P2 about the meal plan in general |
| it’s good for my kids, too, especially for a sandwich to take to school. I like that it includes cucumbers; it adds something different compared to just tomato or a different cheese. Normally, when I make a sandwich, I use yellow cheese, but I liked the change of adding cucumber and lettuce. | P2 (about D5 breakfast) |
| The rice is with pasta because that's how I make the soup, but I do it with pasta, that is, I make noodles so it feels heavier. I felt it this time it is light and maybe because as it is also measured the amount I consumed then it felt very light, that is, I did not feel heavy when I ate it. | P2 (about D1 dinner) |
| Because I really liked how the chicken tasted with the vegetables. I had never made it that way. I usually make omelettes like this, but with dried meat. | P3 (about D2 breakfast) |
| I think it’s something that does me good because it has vegetables. | P4 (about D1 dinner) |
| I liked the vegetables, which were more than the chicken. What I like is that it gives me an alternative to eat rice. | P6 (about D2 lunch) |
| **Exposed ways of reducing fat and substituting red meats in familiar meals** | |
| I always make it, but I usually fry it. This time it was baked, and it turned out differently, but it was delicious.  I liked that there was no oil. I really liked it. | P2 (about D6 lunch) |
| As I mentioned, I liked the potatoes, and the sandwich, well, it’s a regular sandwich because I prepare sandwiches, and it’s always similar. Everything I used is what I always use to make sandwiches. So I feel that something different in this recipe was the potatoes because I’m used to eating potatoes that are fried in oil. But these were made in the air fryer. So it felt different. I thought they would have a different taste, but the potatoes turned out delicious. | P2 (about D6 dinner) |
| I liked it; it was tasty. I realize that sometimes when you use a lot of oil and don’t use it, you feel the difference because the tortillas—I liked how crunchy they turned out. I made them in the air fryer, so I liked it, and I’ve tried other dishes where I’ve seen a lot of oil around. So I liked the preparation of these chilaquiles. | P2 (about D7 breakfast) |
| It’s something new for me. This chile is different because it didn’t have egg and wasn’t fried in oil. . . Well, the idea is to make the recipe healthier by using less oil. Since this was the first time I made stuffed chiles like this in the oven, they turned out different, but I liked the flavor. | P2 (about D7 dinner) |
| Well, it’s tasty, and also [with this recipe] I can eat less red meat and make a chicken picadillo . . . it’s better to eat chicken and fish. So I can change one meal of the week from beef to chicken and make chicken picadillo tostadas instead of beef picadillo, which is how I usually make them when I prepare picadillo tostadas. | P3 (about D4 lunch) |
| I feel it’s healthy. It doesn’t feel greasy. I don’t feel any sugar in it. I like [this version] because normally I make chilaquiles with a piece of corn, or sweet potato, or whatever potatoes I have. You know that’s pure starch and sugar. | P5 (about D7 breakfast) |
| I had never eaten flautas like this, baked, so I wasn’t sure how it would turn out; but I liked the way it turned out. | P6 (about D5 lunch) |
