# Supplementary figures and images for: Applying user-centered design to develop a culturally sensitive, low-calorie meal plan for enhancing dietary behavioral control in MASLD
Source: BMC Nutr. 2026 May 6;12:123. doi: 10.1186/s40795-026-01347-8 (PMC13312602; doi:10.1186/s40795-026-01347-8)

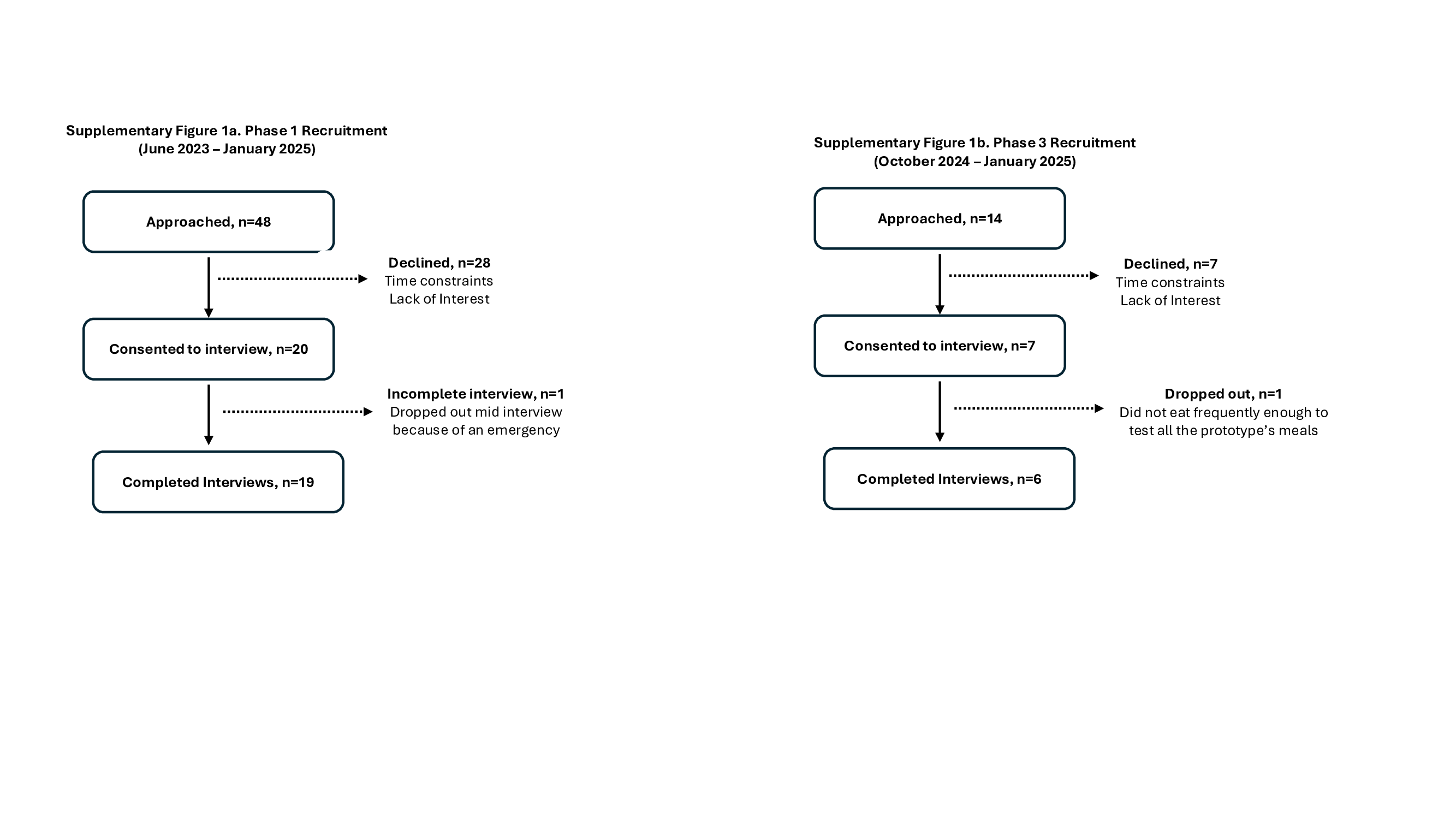

Supplement: Supplementary file 7 — Supplementary Material 7. [file 40795_2026_1347_MOESM7_ESM.tiff]
