# Supplementary material for: Applying user-centered design to develop a culturally sensitive, low-calorie meal plan for enhancing dietary behavioral control in MASLD
Source: BMC Nutr. 2026 May 6;12:123. doi: 10.1186/s40795-026-01347-8 (PMC13312602; doi:10.1186/s40795-026-01347-8)
Supplement: Supplementary file 8 — Supplementary Material 8. [file 40795_2026_1347_MOESM8_ESM.docx]

# **APPENDIX A**

# **Phase 1 - Pre-Interview Survey**

Demographic Questions:

- Where were you born? At what age did you move to the US?
- What is your marital status?
- Do you have children? How many?
- How many people live in your household including yourself?
- What is your occupation or job?

Acculturation questions:

- What language do you most often speak at home?
- What about with your friends?
- What language do you most often read in?
- What language do you think in?

Food insecurity questions

- A lot of people have a hard time affording food, so we routinely ask patients some questions about this. Please let me know if either of these statements is true for you and your family:
  - “We worried whether our food would run out before we got money to buy more.” Was that often true, sometimes true, or never true for (you/your household) in the last 12 months?
  - "We worried the food we bought just didn’t last and we didn’t have money to get more.” Was that often true, sometimes true, or never true for (you/your household) in the last 12 months?

Food shopping and preparation questions

- Where do you go grocery shopping?
- What is your average weekly grocery bill
- Who does the shopping?
- Who does the cooking?

**PHASE 1 INTERVIEW GUIDE**

Thank you for participating in this study interview. We are interviewing patients to learn about their typical eating habits and meals. The purpose is to collect information to develop a healthy meal plan that patients can use to help them lose weight.

For this interview, I will be asking you some questions about your regular diet. I will also ask you to give me as much detail as you can about the food you eat and how it is prepared. You can skip any questions that you do not want to answer.

1. I’d like to know about your typical eating habits. Tell me about where you eat most of your meals, when you eat them, and with whom you eat them.

Weekdays

1. On a typical weekday (let’s say Monday), what is the first thing you have after waking up?

Probes

- How do you prepare it? What method (i.e. frying, baking, boiling, what appliance)? What oil?
- OR where do you buy it from? What does it have?
- How much (amount) do you cook/how big are the portions on your plate?
- What do you add? Do you season it?
- What do you drink?

1. What do you have afterwards?
   1. How do you make it/where do you buy it?
2. On a typical weekday, what do you have for lunch?
   1. Probes:
      1. How do you prepare it? What method (i.e. frying, baking, boiling, what appliance)? What oil?
      2. OR where do you buy it from? What does it have?
      3. How much (amount) do you cook/how big are the portions on your plate?
      4. What do you add? Do you season it?
      5. What do you drink?
3. What do you have afterwards?
   1. How do you make it/where do you buy it?
   2. Do you get hungry between meals?
4. On a typical weekday, what do you have for dinner?
   1. Probes:
      1. How do you prepare it? What method (i.e. frying, baking, boiling, what appliance)? What oil?
      2. OR where do you buy it from? What does it have?
      3. How much (amount) do you cook/how big are the portions on your plate?
      4. What do you add? Do you season it?
      5. What do you drink? Do you prepare it? How?
5. On a typical weekday (let’s say Wednesday), what is the first thing you have after waking up?
   1. Probes:
      1. How do you prepare it? What method (i.e. frying, baking, boiling, what appliance)? What oil?
      2. OR where do you buy it from? What does it have?
      3. How much (amount) do you cook/how big are the portions on your plate?
      4. What do you add? Do you season it?
      5. What do you drink? Do you prepare it? How?
6. What do you have afterwards?
   1. How do you make it/where do you buy it?
7. On a typical weekday, what do you have for lunch?
   1. Probes:
      1. How do you prepare it? What method (i.e. frying, baking, boiling, what appliance)? What oil?
      2. OR where do you buy it from? What does it have?
      3. How much (amount) do you cook/how big are the portions on your plate?
      4. What do you add? Do you season it?
      5. What do you drink? Do you prepare it? How?
8. What do you have afterwards?
   1. How do you make it/where do you buy it?
   2. Do you get hungry between meals?
9. On a typical weekday, what do you have for dinner?
   1. Probes:
      1. How do you prepare it? What method (i.e. frying, baking, boiling, what appliance)? What oil?
      2. OR where do you buy it from? What does it have?
      3. How much (amount) do you cook/how big are the portions on your plate?
      4. What do you add? Do you season it?
      5. What do you drink? Do you prepare it? How?
10. These meals you’ve mentioned, are they your typical meals or out of the ordinary?
    1. IF NOT TYPICAL: What do you more commonly eat? (proceed to probes)
11. These meals you’ve mentioned, are they your preferred meals to have, or do you have them for some other reason? (I.e. time, cost, etc.)
    1. IF NO: what would you have instead? (proceed to probes)

Weekends

1. On a typical Saturday, what is the first thing you have after waking up?
   1. Probes:
      1. How do you prepare it? What method (i.e. frying, baking, boiling, what appliance)? What oil?
      2. OR where do you go/buy it from? What does it have?
      3. How much (amount) do you cook/how big are the portions on your plate?
      4. What do you add? Do you season it?
      5. What do you drink? Do you prepare it? How?
2. What do you have afterwards?
   1. How do you make it/where do you buy it?
3. What do you have for lunch?
   1. Probes:
      1. How do you prepare it? What method (i.e. frying, baking, boiling, what appliance)? What oil?
      2. OR where do you buy it from? What does it have?
      3. How much (amount) do you cook/how big are the portions on your plate?
      4. What do you add? Do you season it?
      5. What do you drink? Do you prepare it? How?
4. What do you have for dinner?
   1. Probes:
      1. How do you prepare it? What method (i.e. frying, baking, boiling, what appliance)? What oil?
      2. OR where do you buy it from? What does it have?
      3. How much (amount) do you cook/how big are the portions on your plate?
      4. What do you add? Do you season it?
      5. What do you drink? Do you prepare it? How?
5. What about Sunday? What is the first thing you have after waking up?
   1. Probes:
      1. How do you prepare it? What method (i.e. frying, baking, boiling, what appliance)? What oil?
      2. OR where do you go/buy it from? What does it have?
      3. How much (amount) do you cook/how big are the portions on your plate?
      4. What do you add? Do you season it?
      5. What do you drink? Do you prepare it? How?
6. What do you have afterwards?
   1. How do you make it/where do you buy it?
7. What do you have for lunch?
   1. Probes:
      1. How do you prepare it? What method (i.e. frying, baking, boiling, what appliance)? What oil?
      2. OR where do you buy it from? What does it have?
      3. How much (amount) do you cook/how big are the portions on your plate?
      4. What do you add? Do you season it?
      5. What do you drink? Do you prepare it? How?
8. What do you have afterwards?
   1. How do you make it/where do you buy it?
   2. Do you get hungry between meals?
9. What do you have for dinner?
   1. Probes:
      1. How do you prepare it? What method (i.e. frying, baking, boiling, what appliance)? What oil?
      2. OR where do you buy it from? What does it have?
      3. How much (amount) do you cook/how big are the portions on your plate?
      4. What do you add? Do you season it?
      5. What do you drink? Do you prepare it? How?
10. These meals you’ve mentioned, are they your typical meals or out of the ordinary?
    1. IF NOT TYPICAL: What do you more commonly eat? (proceed to probes)
11. These meals you’ve mentioned, are they your preferred meals to have?
    1. IF NO: what would you have instead? (proceed to probes)
12. When you go out to eat, where do you typically go or order from?
    1. Probe: what day of the week/time of day do you usually order out?
13. Do you eat dessert? What do you eat? When do you eat it (time of day, day of the week)?
14. What is the most difficult meal of the day or week for you to plan/prepare?
    1. Why?

Staple Foods

1. How do you typically cook your meals? Or do you order/eat out?
   1. Probes:
      1. What oil do you use?
      2. What appliances do you use?
      3. What do you use for seasoning/flavor?

I’d like to know what are typical foods and ingredients you have at home. Think about what you typically shop for and have at home:

1. What are some fruits and veggies you typically have at home?
   1. What about beans? What kind? How do you prepare them?
2. What about grains? Ex: rice, quinoa, bread, tortillas.
   1. When/with what meals do you eat them?
   2. How much/many?
3. What about meat? What kind?
   1. What cut? How much fat; do you know the fat percent or ration?
   2. How do you prepare it?
   3. Probe: what about chicken, fish, seafood?
4. How do you typically season your food?
   1. What do you use to add flavor?
   2. Are there any particular brands you purchase?
   3. Do you use these regularly or just occasionally? Why?
   4. (If they mention they make their own seasoning at home) How do you prepare it?

Wrap Up:

That concludes our interview. Thank you so much for talking with me today and sharing all this information. We’ve covered a lot of territory today, but is there anything else you’d like to share with me about your dietary habits and the foods you prepare and consume regularly?

# **APPENDIX B**

# **Phase 3 - Ecological Momentary Assessment Based Post-Meal Survey**

- - - 1. **For how many people did you prepare the meal?** (E.g. If you prepared the meal for yourself + 3 family members, you should answer 4) / ***¿Para cuántas personas preparo la comida****? (Ej. Si preparó la comida para usted + 3 miembros de la familia, debe responder 4)*
    1. 1
    2. 2
    3. 3
    4. 4
    5. 5+

1. **Which version of the recipe did you use? /*¿Qué versión de la receta uso?***
   - 1. Good for 1 Serving / *Bueno para 1 porción*
     2. Good for 4 Servings / *Bueno para 4 porciones*
     3. Other / *Otro*
2. **Did the recipe require you to purchase any ingredients that you do not typically buy or have in your home?** *¿La receta requería que comprara algunos ingredientes que normalmente no compra o tiene en su casa?*
   1. I had everything I needed at home to make the recipe / *Tenía todo lo que necesitaba en casa para hacer la receta*
   2. The recipe required ingredients I do not typically have at home or buy */ La receta requería ingredientes que normalmente no tengo en casa o que compro*
3. **How long did it take to prepare the entire meal?** (Think about how long it took you to prepare the meal, from the moment you started preparing the ingredients to cooking completion.) /*¿Cuánto tiempo se tardó en preparar toda la comida? (Piensa en cuánto tiempo se llevó en preparar la comida, desde el momento en que comenzó a preparar los ingredientes hasta que terminó de cocinar.)*
   - - Free text response, allowing user to type number of minutes
4. **How much do you think the meal cost**? (Think about the ingredients and the quantities of these various ingredients you used to prepare this meal.) / *¿Cuánto crees que cuesta la comida? (Piense en los ingredientes y las cantidades de estos varios ingredientes que usaste para preparar esta comida.)*
   - Free text response
5. **Did you make any changes to the recipe? / *¿Hizo algún cambio en la receta?***
   - 1. No, I followed it exactly as instructed / *No, lo seguí exactamente como me indicaron*
     2. Yes, I made one or more changes to the recipe / *Sí, hice uno o más cambios a la receta*
6. **Did the recipe work as** **written? / *¿Funcionó la receta como está escrita?***
   - 1. Yes, the recipe worked as written / *Sí, la receta funcionó como está escrita*
     2. No, I had to make changes to the written recipe to make it work / *No, tuve que hacer cambios en la receta escrita para que funcionara*

**Please consider each statement below and rate how strongly you agree or disagree, 1 being strongly disagree and 5 being strongly agree.** *Por favor considere cada frase y califique cuánto está de acuerdo o en desacuerdo, 1 siendo totalmente en desacuerdo y 5 siendo totalmente de acuerdo.*

1. **The cost of the meal was acceptable. / El costo de la comida fue acceptable.**
2. Strongly Disagree / Totalmente en Desacuerdo
3. Disagree / En Desacuerdo
4. Neutral / Neutro
5. Agree / De Acuerdo
6. Strongly Agree / Totalmente de Acuerdo
7. Don’t Know / No Sé
8. **The meal tasted good. / La comida sabía bien.**
9. Strongly Disagree / Totalmente en Desacuerdo
10. Disagree / En Desacuerdo
11. Neutral / Neutro
12. Agree / De Acuerdo
13. Strongly Agree / Totalmente de Acuerdo
14. Don’t Know / No Sé
15. **I would use this recipe often. / Usaría esta receta frecuentemente.**

1) Strongly Disagree / Totalmente en Desacuerdo

2) Disagree / En Desacuerdo

3) Neutral / Neutro

4) Agree / De Acuerdo

5) Strongly Agree / Totalmente de Acuerdo

6) Don’t Know / No Sé

1. **The recipe was easy to use. / La receta era fácil de usar.**
2. Strongly Disagree / Totalmente en Desacuerdo
3. Disagree / En Desacuerdo
4. Neutral / Neutro
5. Agree / De Acuerdo
6. Strongly Agree / Totalmente de Acuerdo
7. Don’t Know / No Sé

**PHASE 3 - INTERVIEW GUIDE**

"Hello [Insert Patient Name]. Hope your day is going well! You've officially completed day [Insert day of the meal plan] of the 7-day meal plan. Now that the day is over, I would like to have a conversation with you and get some feedback regarding the meals. Questions would include how you felt about each meal, how we can improve each one, and other similar questions.

*Check post-meal survey responses and mark which meals they’ve completed for the day & proceed accordingly. Confirm with the participant.*

**Just to confirm, which meals did you prepare and eat today?**

- Breakfast, Day X
- Lunch , Day X
- Dinner, Day X
- Snack, Day X
- None of the above

**GENERAL QUESTIONS ASKED ABOUT ALL MEALS**

1. **Overall, how do you feel about [THE MEAL]?**

Probe:

- What was good about it? (Alternate wording: what did you like about it?)
- What was not good about it? (Alternate wording: what did you not like about it?)

1. **How did you feel the meal tasted?**
2. **How could we improve the meal?** Probe: taste, cost, time to prepare/convenience
3. **I’d like to review the written recipe with you. Tell me how it was following the recipe exactly as written.**

Probe and prompts

- - How understandable was it? What was difficult to understand?
  - How could we improve the written recipe to make it more understandable?
  - As you prepared the meal, what kinds of changes did you have to make to the written recipe?
  - Why did you make the changes?
  - What kind of changes would you make in this recipe? - Why?

1. **Did the recipe call for ingredients that you do not typically have or buy?**

Probes and prompts:

- What were they (the ingredients in the recipe that you don’t typically have at home and/or typically buy?
- how did you change/manage the recipe if you didn’t have the ingredient (did you buy it, find a substitute, cook without it, change the recipe somehow)?
- Tell me more about how you prepared the recipe (e.g. serving size used, etc.)

1. **Tell me about how long it took to prepare the recipe**

- **(**Verify survey reported information).
- Probe acceptability.

1. **Tell me about the cost of the recipe (Verify survey reported information).** Probe acceptability.
2. **Would you use this recipe again?**

Probe:

- Why or why not?
- If person responds no, what would have to change for you to use this recipe more often?

1. *For meals that the participant did not prepare, ask:* **I noticed you did not eat [meal], why is that?** Probe: Is there anything specific that made it difficult for you to eat [meal]?

**FINAL INTERVIEW - QUESTIONS ADDRESSING MEAL PLAN IMPACT**

1. I’d like to know more about your experience following this meal plan over the last week. If you had to prepare these meals week after week, how would you feel?

- Probe: what aspects of the meal plan or recipes were difficult? What was helpful?

1. How has your perspective on healthy eating changed with these meals?
2. By doing this meal plan over the last week, how do you think it will impact your eating routine in the future?
